# Deep-learning systems for diagnosing cleft palate on panoramic radiographs in patients with cleft alveolus

**DOI:** 10.1007/s11282-022-00644-9

**Published:** 2022-08-19

**Authors:** Chiaki Kuwada, Yoshiko Ariji, Yoshitaka Kise, Motoki Fukuda, Masako Nishiyama, Takuma Funakoshi, Rihoko Takeuchi, Airi Sana, Norinaga Kojima, Eiichiro Ariji

**Affiliations:** 1 Department of Oral and Maxillofacial Radiology, Aichi-Gakuin University School of Dentistry, 2-11 Suemori-dori, Chikusa-ku, Nagoya, Japan; 2 Department of Oral Radiology, Osaka Dental University, 5-17, Otemae 1-chome, Chuo-ku, Osaka, Japan; 3 Department of General Dentistry, Dental Hospital, Aichi-Gakuin University School of Dentistry, 2-11 Suemori-dori, Chikusa-ku, Nagoya, Japan

**Keywords:** Deep learning, Panoramic radiography, Cleft palate

## Abstract

**Objectives:**

The aim of the present study was to create effective deep learning-based models for diagnosing the presence or absence of cleft palate (CP) in patients with unilateral or bilateral cleft alveolus (CA) on panoramic radiographs.

**Methods:**

The panoramic images of 491 patients who had unilateral or bilateral cleft alveolus were used to create two models. Model A, which detects the upper incisor area on panoramic radiographs and classifies the areas into the presence or absence of CP, was created using both object detection and classification functions of DetectNet. Using the same data for developing Model A, Model B, which directly classifies the presence or absence of CP on panoramic radiographs, was created using classification function of VGG-16. The performances of both models were evaluated with the same test data and compared with those of two radiologists.

**Results:**

The recall, precision, and F-measure were all 1.00 in Model A. The area under the receiver operating characteristic curve (AUC) values were 0.95, 0.93, 0.70, and 0.63 for Model A, Model B, and the radiologists, respectively. The AUCs of the models were significantly higher than those of the radiologists.

**Conclusions:**

The deep learning-based models developed in the present study have potential for use in supporting observer interpretations of the presence of cleft palate on panoramic radiographs.

## Introduction

The deep learning (DL) algorithm based on a convolution neural network has recently drawn the attention of many researchers and has been applied in many computer-aided diagnosis/detection (CAD) systems including panoramic radiographic diagnosis [1–15]. In many reports on panoramic radiographs, the performance of CAD systems is reported to be superior to that of inexperienced observers and equivalent to that of experienced radiologists [1], sometimes even exceeding the performance of experienced radiologists [2]. An important role of such CAD systems may be to reduce the load on experienced radiologists, who must routinely interpret a large number of images in clinics while supporting inexperienced observers to ensure that they avoid overlooking critical lesions. In such cases, the pathologies on panoramic radiographs, such as mesiodens [2–4], mandibular radiolucent lesions [5], and submandibular sialoliths [6], which can easily be diagnosed by specialists, should be considered as target lesions. Moreover, lesions such as cleft lip and palate (CLP) that can easily be diagnosed by physical examination should also be included because oral and maxillofacial radiologists cannot always perform such examinations and are forced to interpret findings using panoramic radiographs alone.

Cleft lip and palate are one of the most common types of congenital craniofacial anomalies with approximately 1 case per 700 live births [16–18]. Although various classifications have been proposed [19], they are fundamentally based on the status of the CA and cleft palate (CP). The presence or absence of CA is an essential factor of the patient, and when CA is present, whether it is a unilateral or bilateral occurrence should be determined. In our previous study [1], a DL-based CAD system was created to detect CAs on panoramic radiographs regardless of unilateral or bilateral occurrence and the presence or absence of CP. As a result, high performance was achieved, with a recall of 0.88, precision of 0.98, and F-measure of 0.92. As for CPs, however, only one study has reported the performance of a DL-based CAD system to detect the CPs occurring concomitantly with the unilateral CA, and its performance was poor (a recall of 0.67) [2]. Therefore, it has not been confirmed whether a DL-based CAD system can determine the presence of CP in patients with CAs regardless of whether the CA is a unilateral or bilateral occurrence.

The aim of the present study was to create effective DL-based models for diagnosing the presence or absence of CP in patients with unilateral and bilateral CA on panoramic radiographs. This study also evaluated the performances of the proposed models. To achieve this aim, we created two models using two convolutional neural networks and compared their performances with those of human observers.

## Materials and methods

This study was approved by the ethics committee of our university (No. 496) and was performed in accordance with the Declaration of Helsinki.

### Patients

Panoramic images of 491 patients (214 females and 277 males) with a mean age of 8.8 years who had unilateral or bilateral CA were selected from the image database at Aichi-Gakuin University Dental Hospital. The images were collected between August 2004 and July 2020. Images obtained just before bone graft surgery for CA were used for the analysis. Among the 491 patients, 299 patients had CA accompanied by CP and were assigned to the “CP present group”. The remaining 192 patients, who only had CA, were assigned to the “CP absent group”. In the CP present group, 209 and 90 patients had unilateral and bilateral CA, respectively, whereas 174 and 18 patients had unilateral and bilateral CA, respectively, in the CP absent group. The presence of CP was confirmed by medical records and examination of computed tomography images. When the cleft was limited anteriorly to the incisive foramen on the most inferior axial computed tomography slice in which the foramen was visible, the case was assigned to the CP absent group; and when the cleft was extended posteriorly to the incisive foramen, it was assigned to the CP present group. The panoramic images were obtained using a Veraviewepocs unit (J. Morita Mfg. Corp., Kyoto, Japan), with a tube voltage of 75 kV, tube current of 8 mA, and exposure time of 16.2 s, or an AUTO III NTR unit (Asahi Roentgen Industry, Kyoto, Japan), with a tube voltage of 75 kV, tube current of 12 mA, and exposure time of 12 s.

### DL architecture

We created two models (Models A and B) in the present study. Model A was created using a DetectNet, with both object detection and classification functions. This network has five main parts: (1) data input and data augmentation; (2) a fully convolutional network, which extracts features and predicts object classes and bounding boxes per grid square; (3) loss function measurement; (4) bounding box clustering; and (5) mean average precision calculation [5]. The adaptive moment estimation (Adam) solver was used with 0.0001 as the base learning rate. Model B was created using a VGG-16 [20], which has only the classification function. These systems were created on a system running Ubuntu OS version 16.04.2 with an 11 GB graphics processor unit (NVIDIA GeForce GTX 1080 Ti; NVIDIA, Santa Clara, CA, USA). The VGG-16 and customized DetectNet were from the DIGITS library version 5.0 (NVIDIA; https://developer.ndivia.com/digits) and used in the Caffe framework.

### Development and assessment of Model A

**Fig. 1:**
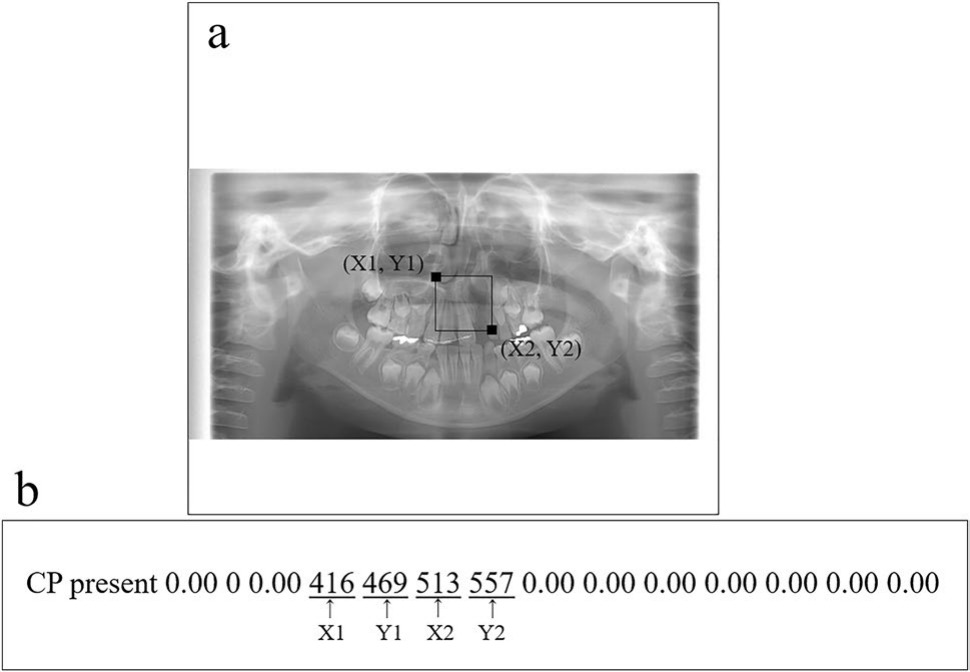
Region of interest (ROI) of the upper incisor area (**a**). The bilateral superior distal ends of the ROI are set at the most distal part of the lateral walls of the nasal cavities. In this case, the vertical position of the right side, which is positioned higher than that of the left side, is chosen as the superior distal end. The inferior margin is set at the alveolar ridge between the central incisors. The coordinates of the two corners are then recorded (**b**)

The panoramic images including whole area of the maxilla and mandible were downloaded in JPEG format and were 900 × 900 pixels in size (Fig. 1a). The datasets used in the learning and inference processes are shown in Table 1. Thirty images were randomly assigned to the test dataset and included both CP present and absent group images. In the CP absent group, only five bilateral CA images were assigned because of the small number of cases. The remaining images not assigned to the test dataset were used as training and validation data for creating the model. The training and validation data were arbitrarily selected using a ratio of approximately 80:20. Model A was created to initially detect the upper incisor area regardless of whether CP was present or absent, and thereafter, the areas were classified into two classes, namely, CP present or absent areas. The upper incisor area, where the CP actually existed or would arise, was defined as a rectangular region of interest (ROI). The bilateral superior distal ends of the ROI were set at the most distal part of the lateral walls of the nasal cavities. When the vertical position differed between the left and right sides, the higher position was chosen as the superior distal end. The inferior margin was set at the alveolar ridge between the central incisors. The coordinates of the upper left (*x*1, *y*1) and lower right (*x*2, *y*2) corners of these ROIs were recorded using ImageJ software (National Institute of Health, Bethesda, MD, USA), and they were converted to text form together with their classifications (CP present or absent; Fig. 1).

**Table 1:** Number of image assignment

	Training data	Validating data	Testing data	Total
CP present group	215 (60)	54 (15)	30 (15)	299 (90)
CP absent group	129 (10)	33 (3)	30 (5)	192 (18)
Total	344 (70)	87 (18)	60 (20)	491 (108)

The number in parenthesis denotes the number of images with bilateral cleft alveolus

**Fig. 2:**
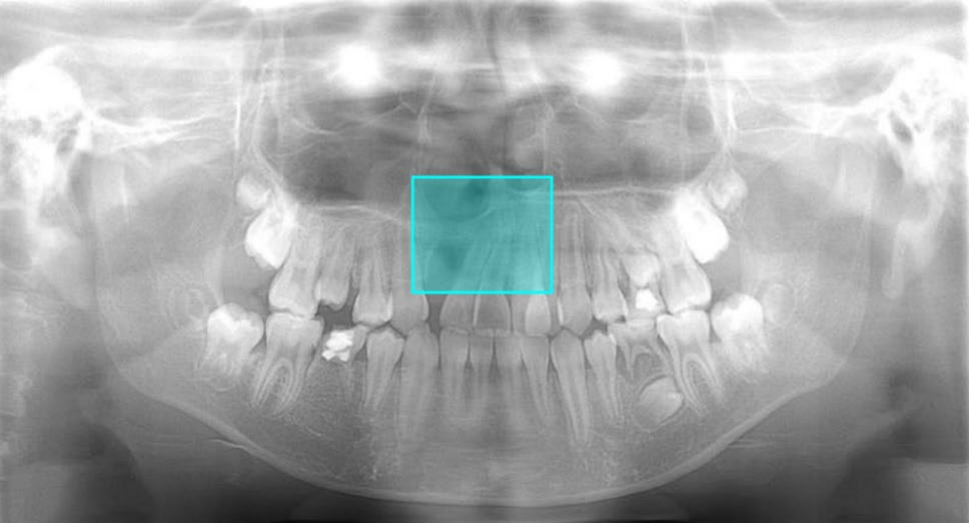
Example of a cleft palate (CP) present image. Model A correctly detected the incisor area and classified it as a CP present area, indicated by the blue box. This case was also correctly classified as a CP present image by Model B

**Fig. 3:**
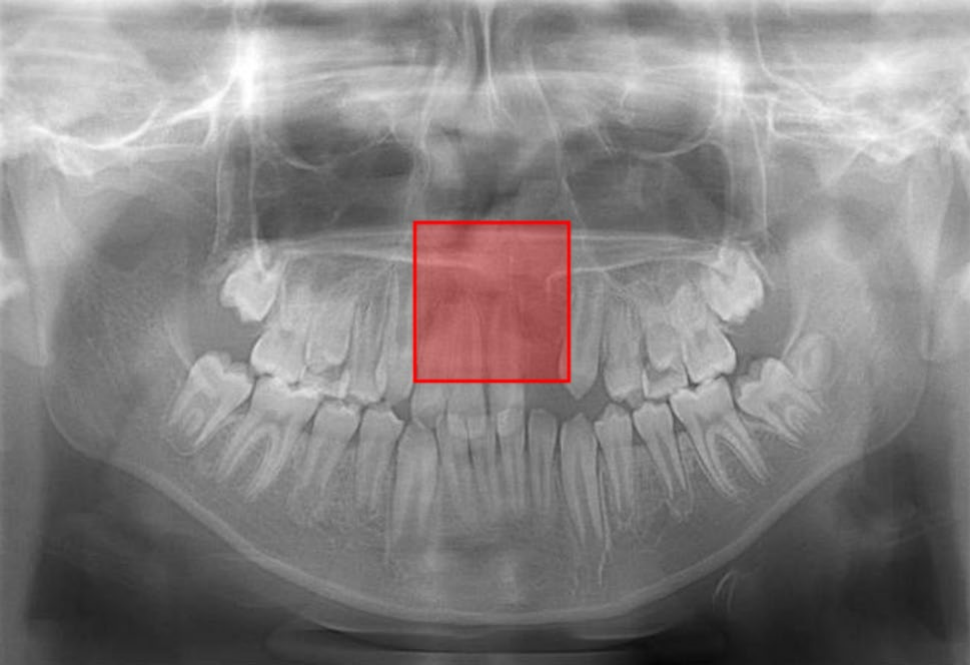
Example of a cleft palate (CP) absent image. Model A correctly detected the incisor area and classified it as a CP absent area (red box). However, Model B falsely classified this image as a CP present image

**Fig. 4:**
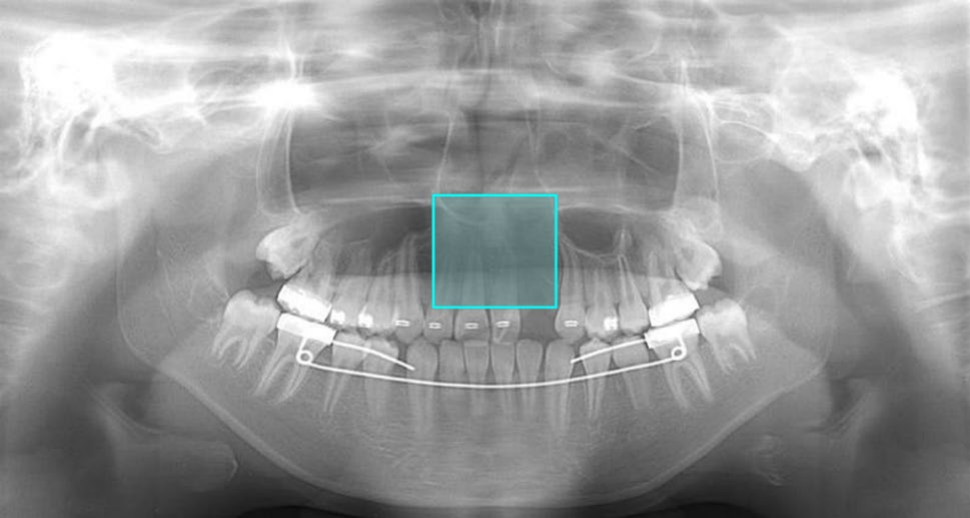
Example of a cleft palate (CP) absent image. Model A correctly detected the incisor area but falsely classified it as a CP present area, as indicated by the blue box. Model B also classified this image incorrectly as a CP present image

When the test data were given to the DL-based model, it predicted a rectangular box showing the incisor area. When the model classified the area as CP present, the box was colored blue, whereas it was red for CP absent areas (Figs. 2,3,4). A box was considered correctly detected when it sufficiently included the location where CAs actually existed or would arise and was limited to the upper incisor area, meaning that the lateral ends did not extend beyond the canine, the superior end did not extend beyond the orbital floor, and the inferior end did not extend beyond the tip of the central incisor.

The detection performance of the incisor area was evaluated using recall, precision, and F-measure, which are defined as follows:

- Recall = number of correctly detected upper incisor areas/number of all upper incisor areas.
- Precision = number of correctly detected upper incisor areas/(number of correctly detected upper incisor areas + number of falsely-detected areas).
- F-measure = 2 (recall × precision)/(recall + precision).

The classification performance for correctly detected upper incisor areas was evaluated by calculating the sensitivity, specificity, accuracy, and the area under the receiver operating characteristic curve (AUC) with the CP present areas considered to be the positive class.

### Development and assessment of Model B

Using the same data used to develop Model A (Table 1), Model B was created for directly classifying the panoramic images into two categories, namely CP present or absent images.

The training data were augmented to create 2600 images by adjusting image sharpness, brightness, and contrast using Irfan View software (Irfan Škiljan, Austria; https://www.irfanview.com/). The learning process was performed in 100 epochs. Thereafter, the test images were input to the developed model, which classified them as CP present or absent images. The classification performance was assessed by calculating sensitivity, specificity, accuracy, and the AUC, with CP present images considered to be the positive class.

### Comparison of DL-based model and human-observer classification performance

To compare the classification performances of the models with those of the human observers, two radiologists with 5 and 6 years of experience diagnosed the same test data used in the assessment of DL-based models. They were asked to classify them into one of two categories (CP present or absent).

### Statistical analysis

The differences between the AUC values of the two models and human observers were statistically assessed using the *χ*^2^ test. The significance level was set to *p* < 0.05.

## Results

Model A correctly detected the upper incisor areas on all 60 test images, and hence the recall, precision, and F-measure were all 1.00. Therefore, the classification performance could be evaluated using the same images for both models and observers, and we could statistically compare their AUC values.

The classification performance is summarized in Table 2. Summing the unilateral and bilateral CA data, both models A and B achieved high performance scores. The AUCs were over 0.9 and no difference could be found between them. Comparing the performances of the unilateral and bilateral CA groups, the values were sufficiently high for both groups.

**Table 2:** Classification performance of presence of cleft palate according to the status of cleft alveolus

	Model A	Model B	Radiologist 1	Radiologist 2
Unilateral CA				
Sensitivity	0.93 (14/15)	1.00 (15/15)	0.73 (11/15)	0.53 (8/15)
Specificity	0.96 (24/25)	0.88 (22/25)	0.68 (17/25)	0.80 (20/25)
Accuracy	0.95 (38/40)	0.93 (37/40)	0.70 (28/40)	0.70 (28/40)
Bilateral CA				
Sensitivity	1.00 (15/15)	1.00 (15/15)	0.73 (11/15)	0.46 (7/15)
Specificity	0.80 (4/5)	0.80 (4/5)	0.60 (3/5)	0.60 (3/5)
Accuracy	0.95 (19/20)	0.95 (19/20)	0.70 (14/20)	0.50 (10/20)
Unilateral and bilateral CA				
Sensitivity	0.96 (29/30)	1.00 (30/30)	0.73 (22/30)	0.50 (15/30)
Specificity	0.93 (28/30)	0.86 (26/30)	0.66 (20/30)	0.76 (23/30)
Accuracy	0.95 (57/60)	0.93 (56/60)	0.70 (42/60)	0.63 (38/60)
AUC	0.95^a,b^	0.93^c,d^	0.70^a,c^	0.63^b,d^

*CA* cleft alveolus, *AUC* area under the receiver operating characteristic curve

^a,b,c,d^Values with the same character denote significant difference between them by the Chi-square test with *p* < 0.05

The performance values of the human observers were relatively low, but no difference in AUCs was found between the two radiologists. By contrast, the AUCs obtained by the observers were significantly different from those obtained by both models.

Typical results are shown in Figs. 2,3 and 4.

## Discussion

In previous studies using the DL object detection technique on panoramic radiographs, many authors have tried to directly detect the pathologies, such as radiolucent cyst-like lesions [5, 8, 9], vertical root fracture [10], and sialoliths [6]. The high detection performances of these studies may partially be attributed to the well-defined appearances of these lesions and a sufficient amount of learning data. In contrast, our previous studies on detecting maxillary sinus pathologies and mesiodens, which were first performed with the same direct detection procedures, did not provide successful results. This may be partially due to the difficulty of diagnosing these lesions. The difference in density between an abnormal sinus, especially one with sinusitis, and a healthy sinus cannot always be differentiated completely on panoramic radiographs. The mesiodens may sometimes be obscured depending on its relation to the panoramic image layer. Therefore, anatomical areas including the maxillary sinus [15] and upper incisor regions [7] were detected before classifying the presence or absence of pathologies in such areas. Consequently, almost perfect detections were achieved with recalls of 0.98 and 1.00 for the maxillary sinus and upper incisor regions, respectively, together with high classification accuracies over 0.90. In the present study, therefore, Model A was created using a DetectNet for detecting the upper incisor area on panoramic radiographs, where CP actually existed or would occur, and it simultaneously classified the areas into two categories indicating the presence or absence of CP. As a result, the recall, precision, and F-measure were all 1.0. It may be relatively easy for a DL-based model to learn certain anatomical regions, such as the upper incisor area in the present study, because the coordinates of such regions are always similar on panoramic radiographs.

In the present study, Model B was created because it would be more useful clinically than Model A if it could directly diagnose the presence of CP without the need to detect the upper incisor area. It is generally recommended to use a smaller area for classification to improve performance [21, 22]. However, Model B showed sufficiently high performance (an AUC of 0.93) that is comparable to that of Model A (an AUC of 0.95) with small classification ROIs. A possible reason for this result is that the presence of CP may affect a relatively wide area on panoramic radiographs, indicating that there may be widespread differences in the appearance of cases with and without CP. Accordingly, this might cause the classification performance of Model B to be high.

Comparing the classification performance of the models with those of human observers (oral and maxillofacial radiologists), both models achieved values that were significantly higher than those obtained by the radiologists, who had low AUCs of 0.70 and 0.63. This result may verify the efficacy of a DL-based CAD system for supporting busy radiologists in the interpretation of panoramic radiographs of patients with CLP. In addition, because CP is easy to recognize by physical examination, detailed analyses may not be performed to identify the difference of the panoramic appearances of cases with and without CP, causing findings effective for differentiation to be overlooked.

The present study has some limitations. First, although the quality of a panoramic image can be easily altered depending on the position of the panoramic image layer in the incisor area, almost all radiographs in the present study were taken by experienced technicians and were good quality images. This might have increased the detectability and classification performance. To take the conditions of actual clinical use into account, the performances should be verified on poor quality images. Second, the number of CP absent images was small in patients with bilateral CAs, resulting in a relatively low specificity. Third, the normal subjects were not included in the test data. In the present study, the models were created to classify only the cases with CAs because we had developed a high-performance model for detecting Cas [1]. However, to enable it to be used for screening purposes, normal cases should be included in the test data. Fourth, we did not analyze the differences in imaging findings of the cases with and without CP. Future research should be conducted to investigate these differences. Inconclusion, the DL-based models developed in the present study have potential for use in supporting observer interpretations of the presence of CA on panoramic radiographs.
